# *Sinomonas terrae* sp. nov., Isolated from an Agricultural Soil

**DOI:** 10.4014/jmb.2302.02009

**Published:** 2023-04-13

**Authors:** Hyosun Lee, Ji Yeon Han, Dong-Uk Kim

**Affiliations:** Department of Biological Science, College of Science and Engineering, Sangji University, Wonju 26339, Republic of Korea

**Keywords:** Sinomonas, taxonomy, polyphasic study, novel member

## Abstract

While searching for the bacteria which are responsible for degradation of pesticide in soybean field soil, a novel bacterial strain, designated 5-5^T^, was isolated. The cells of the strain were Gram-staining-positive, aerobic and non-motile rods. Growth occurred at 10-42°C (optimum, 30°C), pH 5.5-9.0 (optimum, pH 7.0-7.5), and 0-2% (w/v) NaCl (optimum, 1%). The predominant fatty acids were C_15:0_ anteiso, C_17:0_ anteiso, and summed feature 8 (C_18:1_ *ω*7*c* and/or C_18:1_ *ω*6*c*). The predominant menaquinone was MK-9 (H_2_). Diphosphatidylglycerol, glycolipids, phosphatidylinositol, and phosphatidylglycerol were the major polar lipids. Phylogenetic analysis of 16S rRNA gene sequences indicated that strain 5-5^T^ is a member of the genus *Sinomonas* and its closest relative is *Sinomonas humi* MUSC 117^T^, sharing a genetic similarity of 98.4%. The draft genome of strain 5-5^T^ was 4,727,205 bp long with an N50 contig of 4,464,284 bp. Genomic DNA G+C content of strain 5-5^T^ was 68.0 mol%. The average nucleotide identity (ANI) values between strain 5-5^T^ and its closest strains *S. humi* MUSC 117^T^ and *S. susongensis* A31^T^ were 87.0, and 84.3 % respectively. In silico DNA-DNA hybridization values between strain 5-5^T^ and its closest strains *S. humi* MUSC 117^T^ and *S. susongensis* A31^T^ were 32.5% and 27.9% respectively. Based on the ANI and *in silico* DNA-DNA hybridization analyses, the 5-5^T^ strain was considered as novel species belonging to the genus *Sinomonas*. On the basis of the results from phenotypic, genotypic and chemotaxonomic analyses, strain 5-5^T^ represents a novel species of the genus *Sinomonas*, for which the name *Sinomonas terrae* sp. nov. is proposed. The type strain is 5-5^T^ (=KCTC 49650^T^=NBRC 115790^T^).

## Introduction

The genus *Sinomonas* was initially introduced by Zhou *et al*. [1] and the genus is a member of the family *Micrococcaceae*. Following the introduction of the first novel species in 2009, there has been an increase in the number of recently discovered species with valid names. As of now, there are 10 species in the genus *Sinomonas* have been validly named and published (https://lpsn.dsmz.de/genus/sinomonas): *S. flava*, *S. atrocyanea*, *S. soli*, *S. echigonensis*, *S. albida*, *S. halotolerans*, *S. humi*, *S. mesophila*, *S. notoginsengisoli*, and *S. susongensis* [1-9]. *Sinomonas* species have been discovered in various environments, including polluted forest soil, surface of weathered biotite, and ancient fort soil. Cells of genus *Sinomonas* are Gram-staining-positive (or variable) and aerobic rods. MK-9(H_2_) is the most abundant menaquinone present in the genus *Sinomonas*. Anteiso-C_17:0_, anteiso-C_15:0_, and iso-C_15:0_, are the predominant fatty acids in the genus *Sinomonas*. Diphosphatidylglycerol, phosphatidylglycerol, phosphatidylinositol, and two glycolipids are the primary polar lipids found in the genus. This study seeks to determine the taxonomic position of strain 5-5^T^ and present supporting evidence indicating that it is a novel species belonging to the *Sinomonas* genus.

## Materials and Methods

### Isolation and Culture Conditions

The isolation of strain 5-5^T^ was accomplished from a soil sample that was gathered on June 11, 2020, from a soybean field located in Gyeonggi-do, Republic of Korea, at the GPS coordinates of 37°12¢10.1¢¢N 127°10¢24.2¢¢E. The strain 5-5^T^ was isolated using the standard dilution plating technique on Peptone–Tryptone–Yeast extracts–Glucose (PTYG) agar. The PTYG medium used had a composition of 0.25 g of peptone (Difco), 0.25 g of tryptone (Difco, USA), 0.5 g of yeast extracts (Difco), 0.5 g of glucose, 0.03 g of MgSO_4_, and 0.003 g of CaCl_2_ per liter. Following the plating procedure, the plates were incubated at 28°C. After 2 days, a creamy-white coloured colonies were isolated and identified as 5-5^T^. To ensure long-term preservation of the strain, the strain 5-5^T^ was stored at -80°C in PTYG broth with 20% (v/v) glycerol. Additionally, the strain was deposited in both Korean Collection for Type Cultures (KCTC) and NITE Biological Resource Center.

### Phylogenetic Analysis

To amplify the partial 16S rRNA gene of strain 5-5^T^, colony PCR was conducted using the forward primer 27F (5¢-AGAGTTTGATCMTGGCTCAG-3¢) and reverse primer 1492R (5¢-GGYTACCTTGTTACGACTT-3¢), which were previously described [10]. Following the PCR amplification of the partial 16S rRNA gene of strain 5-5^T^, the PCR product was purified and sequenced using the 785F and 907R universal bacterial primers. The resulting sequence data was compiled using SeqMan software, which is developed by DNASTAR in the USA. Specifically, the software was used to generate an almost complete sequence of the 16S rRNA gene. To identify the closest phylogenetic species of strain 5-5^T^, the 16S rRNA gene sequence was analyzed using the EzBioCloud server (http://ezbiocloud.net) [11]. To perform the phylogenetic analysis, 16S rRNA gene sequences of the closest relatives were obtained from the GenBank database. The obtained 16S rRNA gene sequences of the related bacteria were aligned through multiple alignment using SILVA alignment tool according to the SILVA seed alignment [12]. To construct the phylogenetic trees, three algorithms were used: neighbor-joining [13], maximum-parsimony [14], and maximum-likelihood [14], using the software MEGA7 [15]. Kimura's two-parameter model was utilized to compute the evolutionary distances between the aligned 16S rRNA gene sequences [16]. Bootstrap analysis was performed using 1,000 replications [17].

### Whole Genome Sequencing, Assembly, and Annotation

For whole genome sequencing, genomic DNA of strain 5-5^T^ was extracted using a Maxwell® Prokaryote/Eukaryote SEV DNA Purification Kit (Promega) according to the manufacturer’s procedure. Whole genome shotgun sequencing was performed using the Illumina HiSeq2500 platform by Macrogen in South Korea. The sequencing data was then assembled using SPAdes version 3.14 [18], and the resulting genome sequence was annotated using the NCBI Prokaryotic Genome Annotation Pipeline (PGAP) [19]. The DNA G+C content of strain 5-5^T^ was determined by analyzing the whole genome sequence. To determine the genome-based relatedness between strain 5-5^T^ and closest type strains, the ANI calculator [20] was used to determine the Average Nucleotide Identity (ANI), and the Genome-to-Genome Distance Calculator (GGDC 2.1; https://ggdc.dsmz.de/ggdc.php)[21] was utilized for *in silico* DNA-DNA hybridization (dDDH) value calculations. These values are commonly used to estimate the genetic relatedness between bacterial strains at the species level.

### Phenotypic Characterization

Cellular morphology of strain 5-5^T^ was determined using transmission electron microscopy (LIBRA 120; Carl Zeiss, Germany). The cells were grown on R2A agar for 2 days at 30°C before being examined. The strain 5-5^T^ was tested for growth on different types of media including Nutrient Agar (NA), Luria-Bertani Agar (LBA), R2A Agar (R2A), Trypticase Soy Agar (TSA), and MacConkey Agar (MCA). All the media were manufactured by BD Difco in the USA. The activities of Catalase and Oxidase of the strain 5-5^T^ were tested using commercial reagents, specifically the ID Color Catalase Reagent and Oxidase Reagent from bioMérieux, France. The strain 5-5^T^ was subjected to tests for its ability to grow at various temperatures, pH levels, and NaCl concentrations in R2A broth after 7 days of incubation at 30°C. To determine the temperature range suitable for growth, the strain was subjected to growth testing at various temperatures ranging from 4°C to 45°C. The temperatures tested were 4, 10, 18, 25, 30, 37, 42, and 45°C. The pH range for growth was determined by adjusting the pH of the R2A broth to 4.0-10.0 with citrate buffer (pH 4.0-5.5), phosphate buffer (pH 6.0-7.5), and Tris buffer (pH 8.0-10.0). Tolerance to NaCl was tested in R2A broth with varying concentrations of NaCl (0-10% w/v). To determine the growth of the strain in anaerobic conditions, an anaerobic growth test was conducted by incubating strain 5-5^T^ on R2A agar at 30°C for 14 days using an anaerobic jar and BD GasPak EZ Gas Generating Pouch System. After being incubated for 14 days, the strain's capacity for hydrolyzing casein, starch, DNA, cellulose, and Tween 80 was evaluated according to the Smibert and Krieg's methods [22]. The API ZYM and API 20NE systems (bioMérieux, France) were utilized to test for other physiological and biochemical properties.

### Chemotaxonomic Characterization

To examine the cellular fatty acid composition, a fatty acid methyl ester (FAME) analysis was conducted. The strain 5-5^T^, *Sinomonas humi* NBRC 110653^T^, *Sinomonas susongensis* JCM 31752^T^, *Sinomonas atrocyanea* KACC 17386^T^, and *Sinomonas soli* KCTC 19389^T^ were cultivated on R2A agar at 30°C for 2 days at the same time. After cultivation, the cellular fatty acids were extracted, methylated, and separated using gas chromatography (model 7890; MIDI, USA) following the Sherlock Microbial Identification System's protocol [23]. The identification and quantification of fatty acid methyl esters were performed using the Sherlock Microbial Identification System's TSBA 6 database (version 6.3). After cultivation on R2A agar, the cells were harvested by centrifugation and subjected to freeze-drying for analysis of isoprenoid quinones. The extraction of isoprenoid quinones was performed using a chloroform/methanol solution (2:1, v/v), followed by purification with a Sep-pak kit (Waters, USA) and subsequent analysis via HPLC, as described by Collins and Jones [24]. The freeze-dried cells were used for extracting polar lipids, which were then separated using two-dimensional TLC based on the method developed by Minnikin *et al*. [25]. Individual polar lipids were identified by spraying the plates with 10% ethanolic molybdophosphoric acid for total lipids, molybdenum blue for phospholipids, ninhydrin for amino lipids, *α*-naphthol reagents for glycolipids, and Dragendorff 's reagent for phosphatidylcholine [26].

## Results and Discussion

### Phylogenetic Analysis

**Fig. 1:**
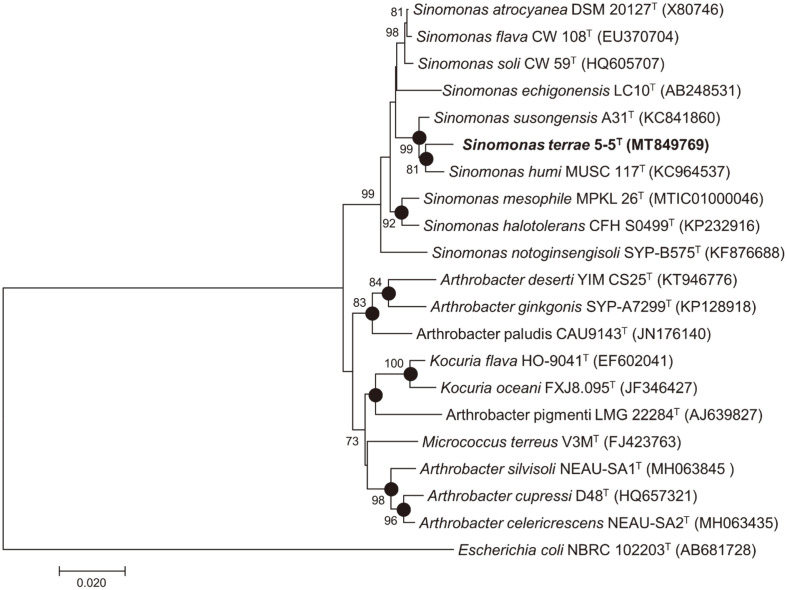
Maximum-likelihood tree based on 16S rRNA gene sequences showing the relationship between strain 5-5^T^ and related species. The dots indicate that the corresponding branches were also recovered in the neighbor-joining tree and maximum parsimony tree. Bootstrap values (expressed as percentages of 1,000 replications) greater than 70% are shown at branch points. *Escherichia coli* NBRC 102203^T^ was used as outgroup. Bar, 0.020 nucleotide substitutions per positions.

Following 16S rRNA gene PCR sequencing, strain 5-5^T^ yielded a 1,460 bp sequence, which was deposited in GenBank under the accession number MT849769. The strain 5-5^T^ belonged to the class Actinomycetia, order Micrococcales, and family *Micrococcaceae*. Hihg sequence similarity was observed with *S. humi* MUSC 117^T^, *S. susongensis* A31^T^, *S. atrocyanea* KCTC 3377^T^, and *S. soli* CW 59^T^ (98.4, 98.4, dk97.3, and 97.2% sequence similarity, respectively). Based on the threshold value of 98.7% similarity for bacterial species delineation [27], the strain 5-5^T^ represents a novel species of the genus *Sinomonas*. Analysis using three different tree-building approaches (neighbor-joining, maximum-parsimony, and maximum-likelihood) indicated that strain 5-5^T^ was closely related to other members of the *Sinomonas* genus (Fig. 1). The closely related type strain *S. humi* NBRC 110653^T^, *S. susongensis* JCM 31752^T^, *S. atrocyanea* KACC 17386^T^, and *S. soli* KCTC 19389^T^ were chosen as reference strains.

### Whole Genome Sequencing, Assembly, and Annotation

The complete genome sequence of strainm5-5^T^ has been submitted to DDBJ/ENA/GenBank and assigned the accession number JAKZBV000000000. The size of the genome was 4,727,205 bp, with an N50 value of 4,464,284 bp (Table 1). The genome of the strains consists 3 contigs with genome coverage of 207.86×. The genome included 4,343 protein-coding genes, 109 pseudo genes, 12 rRNAs, and 55 tRNAs. The G+C content of strain 5-5^T^ was determined to be 68.0 mol%, which falls within the reported range for the genus *Sinomonas* (66.6-71.8 mol%). The ANI values between strain 5-5^T^ and its closest strains *S. humi* MUSC 117^T^, *S. susongensis* A31^T^, and *S. atrocyanea* KCTC 3377^T^ were 87.0%, 84.3%, and 78.0% respectively. The ANI values obtained for strain 5-5^T^ with other *Sinomonas* species were below the threshold of 95% [20]. In silico DDH values between strain 5-5^T^ and its closest strains *S. humi* MUSC 117^T^, *S. susongensis* A31^T^, and *S. atrocyanea* KCTC 3377^T^ were 32.5%, 27.9%, and 21.7%, respectively. These values were found to be below the threshold of 70% [21]. These results suggested that the strain 5-5^T^ exhibits species-level differences from other species of the genus *Sinomonas*.

### Phenotypic Characterization

**Fig. 2:**
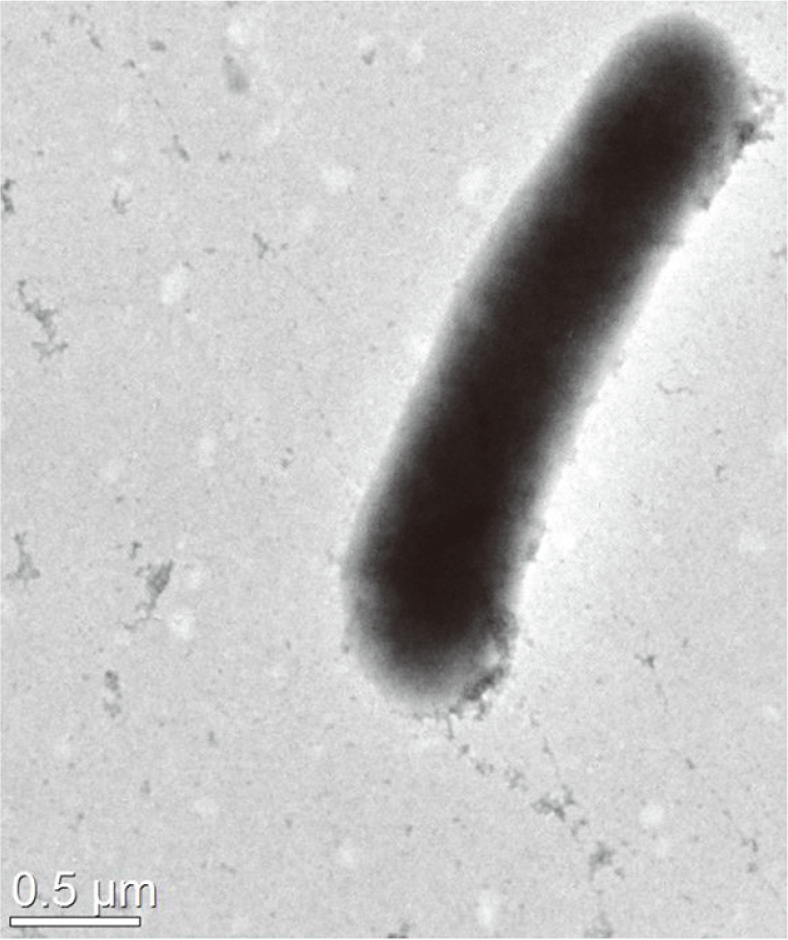
Transmission electron microscopy of strain 5-5^T^. The cells were grown on R2A agar for 2 days at 30°C. Bar, 0.5 μm.

Cells of strain 5-5^T^ were identified as Gram-staining-positive, non-motile, and aerobic rods with 0.6-0.75 × 2.5-3.0 μm in size. (Fig. 2). The strain formed smooth, convex, circular and creamy-white colonies when grown on R2A agar at 30°C for 2 days. Enzyme reaction for oxidase was negative and catalase was positive. Strain 5-5^T^ grew on NA, R2A, LB, TSA, MCA. Strain 5-5^T^ hydrolyzed starch and Tween 80 but could not hydrolyze casein, DNA, and cellulose. The strain 5-5^T^ exhibited a growth range between 10-42 °, pH 5.5-9.0, and 0-2% (w/v) NaCl. The Optimal growth conditions for the strain were observed at 30°C, pH 7.0-7.5, and 1% (w/v) NaCl. The results of physiological and biochemical features of strain 5-5^T^ and other *Sinomonas* species are presented in detail in Table 2.

### Chemotaxonomic Characteristics

**Fig. 3:**
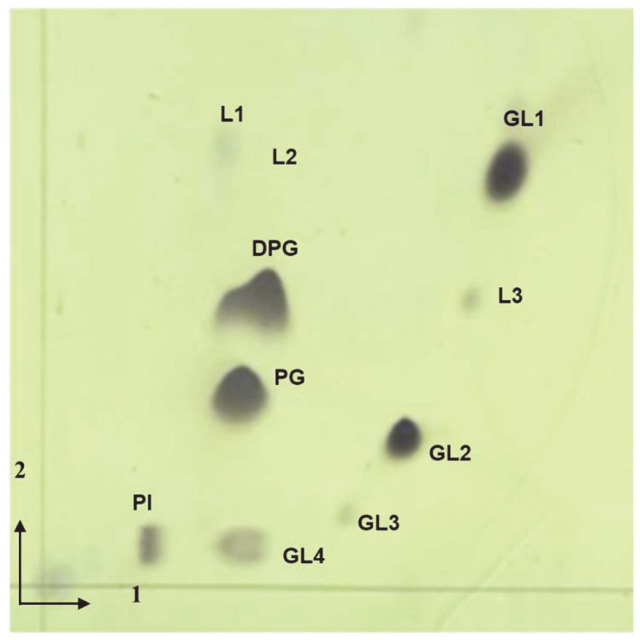
Two-dimensional TLC showing the total polar lipids profile of 5-5^T^. DPG, diphosphatidylglycerol; PG, phosphatidylglycerol; PI, phosphatidylinositol; GL1-4, unidentified glycolipids; L1-3, unidentified lipids.

The fatty acid composition of strain 5-5^T^ and other closely related species in the *Sinomonas* genus are shown in Table 3. Major fatty acids of the strain 5-5^T^ were C_15:0_ anteiso (53.8%) and C_17:0_ anteiso (25.5%). These fatty acids are also present in high amounts in other species of the *Sinomonas* genus such as *S. humi* NBRC 110653^T^, *S. susongensis* JCM 31752^T^, *S. atrocyanea* KACC 17386^T^, and *S. soli* KCTC 19389^T^, indicating a similar fatty acid profile among these species. The polar lipids of strain 5-5^T^ were diphosphatidylglycerol (DPG), phosphatidylglycerol (PG), phosphatidylinositol (PI), four unidentified glycolipids (GL1-4), and three unidentified lipids (L1-3) (Fig. 3). The predominant polar lipids in the strain 5-5^T^ are DPG, PG, PI, and GL. These polar lipids are typical for species within the genus *Sinomonas*. Therefore, the polar lipids pattern support that the strain 5-5^T^ can be affiliated with the genus *Sinomonas*. The dominant menaquinone present in the strain was MK-9(H_2_), which is a typically found in species belonging the genus *Sinomonas*.

The presented finding suggested that the strain 5-5^T^ shares some common characteristics with other species of the genus *Sinomonas*, but also exhibits distinct features that set it apart from them. Therefore, the strain 5-5^T^ is proposed to be a new species within the genus *Sinomonas*, and named as *Sinomonas terrae* sp. nov.

### Description of *Sinomonas terrae* sp. nov.

***Sinomonas terrae* (ter¢rae. L. gen. n. terrae of the soil).** Cells are Gram-stain-positive, non-motile, and aerobic rods with 0.6-0.75 μm in width and 2.5-3.0 μm. The strain formed smooth, convex, circular and creamy-white colonies on R2A agar. The strain is capable of aerobic growth on several types of media, including NA, R2A, LB, TSA, and MCA. Growth occurs at 10-42°C, pH 5.5-9.0, and 0-2% (w/v) NaCl. Optimal growth occurs at 30°C, pH 7.0-7.5, and 1% (w/v) NaCl. Enzyme reaction for oxidase is negative and catalase is positive. Starch and Tween 80 are hydrolyzed but casein, DNA, and cellulose are not hydrolyzed. Nitrate is reduced to nitrite. Esculin is hydrolyzed and gelatin is not hydrolyzed. Indole is not produced and glucose is not fermented. The strain produces *β*-galactosidase, esterase (C4), esterase lipase (C8), leucine arylamidase, acid phosphatase, naphtol-AS-BI-phosphohydrolase, *α*-galactosidase, *β*-galactosidase, *α*-glucosidase, *β*-glucosidase, and *α*-mannosidase. The strain does not produce arginine dihydrolase, urease, alkaline phosphatase, lipase (C14), valine arylamidase, cystine arylamidase, trypsin, *α*-chymotrypsin, *β*-glucuronidase, *N*–acetyl-*β*-glucosaminidase, and *α*-fucosidase. In API 20NE tests, nitrate reduction, *β*-galactosidase, *β*-galactosidase, D-glucose, D-mannose, D-mannitol, D-maltose, gluconate, malic acid, and phenylacetic acid activities are positive, but indole production, glucose fermentation, arginine dihydrolase, urease, protease, L-arabinose, N-acetyl-D-glucosamine, capric acid, adipic acid, and trisodium citrate activities are negative. In API ZYM tests, esterase (C4), esterase lipase (C8), leucine arylamidase, acid phosphatase, naphtol-AS-BI-phosphohydrolase, *α*-galactosidase, *β*-galactosidase, *α*-glucosidase, *β*-glucosidase, and *α*-mannosidase are positive, but alkaline phosphatase, lipase (C14), valine arylamidase, cystine arylamidase, trypsin, *α*-chymotrypsin, *β*-glucuronidase, *N*–acetyl-*β*-glucosaminidase, and *α*-fucosidase activities are negative. The only menaquinone is MK-9(H_2_). The major fatty acids are C_15:0_ anteiso, C_17:0_ anteiso, and summed feature 8 (C_18:1_ *ω*7*c* and/or C_18:1_ *ω*6*c*). The polar lipids are diphosphatidylglycerol, phosphatidylglycerol, phosphatidylinositol, four unidentified glycolipids, and three unidentified lipids. The genomic DNA G+C content is 68.0mol%. The type strain is 5-5^T^ (=KCTC 49650^T^=NBRC 115790^T^), isolated from soybean field soil sample collected from Gyeonggi-do, South Korea (37°12¢10.1"N 127°10¢24.2"E). The 16S rRNA gene and whole genome sequences of strain 5-5^T^ have been assigned the GenBank accession numbers MT849769 and JAKZBV000000000, respectively.

## Figures and Tables

**Table 1:** General genomic feature of strain 5-5^T^.

Genome features	Value
Accession	JAKZBV000000000
Genome size (bp)	4,727,205
G+C content (mol%)	68.0
No. of contigs	3
N50	4,464,284
Total genes	4,413
CDSs (total)	4,343
Genes (RNA)	70
rRNAs (5S, 16S, 23S)	12 (4, 4, 4)
tRNAs	55
ncRNAs	4
Pseudo Genes (total)	109
Genome coverage	207.86×

**Table 2:** Differential phenotypic characteristics of strain 5-5^T^ and type strains of closely related species.

Characteristics	1	2	3	4	5
Assimilation of:					
D-Mannose	+	+	-	+	+
Citrate	-	+	+	-	-
N-acetyl-D-glucosamine	-	-	+	+	+
Enzyme activity of:					
Nitrate reduction	+	-	-	+	+
Arginine dihydrolase	-	-	-	+	-
Urease	-	-	-	+	-
Valine arylamidase	-	-	+	-	-
Cystine arylamidase	-	-	+	-	-
*α*-galactosidase	+	+	+	+	-
*β*-glucuronidase	-	-	-	+	+

Strains: 1, 5-5^T^ (*Sinomonas terrae* sp. nov.); 2, *Sinomonas humi* NBRC 110653^T^; 3, *Sinomonas susongensis* JCM 31752^T^; 4, *Sinomonas atrocyanea* KACC 17386^T^; 5, *Sinomonas soli* KCTC 19389^T^. All data were obtained from this study. All strains are positive for enzyme activity of *β*-glucosidase, *β*-galactosidase, D-glucose, D-mannitol, D-maltose, malic acid, phenylacetic acid, esterase (C4), leucine arylamidase, acid phosphatase, naphtol-AS-BI-phosphohydrolase, *β*-galactosidase, , *α*-glucosidase, *β*- glucosidase, and *α*-mannosidase. All strains are negative for Indole production, L-arabinose, capric acid, adipic acid, alkaline phosphatase, lipase (C14), trypsin, *α*-chymotrypsin, *N*–acetyl-*β*-glucosamine, and *α*-fucosidase. +, Positive; -, negative.

**Table 3:** Cellular fatty acid contents of strain 5-5^T^ and closely related species.

Fatty acid	1	2	3	4	5
Saturated:					
C_16:0_	1.3	2.0	1.1	1.2	tr
Branched-chain fatty acid:					
C_14_:0 iso	tr	tr	tr	tr	1.3
C_15:0_ iso	6.2	13.6	6.6	6.7	13.7
C_16:0_ iso	4.1	4.7	5.8	5.7	5.2
C_17:0_ iso	1.2	2.4	tr	tr	tr
C_15:0_ anteiso	53.8	47.7	64.7	62.4	68.0
C_17:0_ anteiso	25.5	20.6	19.9	21.8	9.5
Summed features:					
8	7.3	8.3	-	-	-

Strains: 1, 5-5^T^ (*Sinomonas terrae* sp. nov.); 2, *Sinomonas humi* NBRC 110653^T^; 3, *Sinomonas susongensis* JCM 31752^T^; 4, *Sinomonas atrocyanea* KACC 17386^T^; 5, *Sinomonas soli* KCTC 19389^T^. All data were obtained from this study. Prior to fatty acid extraction, all strains were grown on R2A agar at 30°C for 2 days. Values are percentages of total fatty acids, and only fatty acids representing more than 1% for at least one of the strains are shown. -, Not detected; tr, trace amounts (<1%). *Summed features represent groups of two or three fatty acids that cannot be separated by gas-liquid chromatography with the MIDI system. Summed feature 8 comprises C_18:1_ *ω*7*c* and/or C_18:1_ *ω*6*c*.
